# Some Dont’s of Our Own

**Published:** 1898-07

**Authors:** 


Some Dont's of Our Own.--*Don't* you, reader, think this "don't" business has been about run in the ground? If you *don't*, I do, and the writers of them ought to be made to don't. (They won't don't). Why *don't* somebody tell a feller what to *do*, and not keep on telling him what to *don't* do.—eh?

